# Coconut oil rubbing as an easy and safe way to improve symptoms in primary nocturnal enuresis: A randomized double-blinded placebo-controlled clinical trial

**DOI:** 10.22038/AJP.2022.20346

**Published:** 2022

**Authors:** Abolfazl Dehghanpour, Monire Seyedhashemi, Ahmad Zare Bidaki, Zohre Mousavi, Majid Emtiazy, Mehrdad Shakiba

**Affiliations:** 1 *Department of Persian Medicine, School of Persian Medicine, Shahid Sadoughi University of Medical Sciences, Ardakan, Yazd, Iran*; 2 *Research Center for Iranian Traditional Medicine, Shahid Sadoughi University of Medical Sciences, Yazd, Iran*; 3 *Department of Pediatrics, Children Growth Disorder Research Center, Shahid Sadoughi University of Medical Sciences, Yazd, Iran*

**Keywords:** Enuresis, Mono symptomatic nocturnal enuresis, Coconut oil, Herbal medicine, Traditional medicine

## Abstract

**Objective:**

Enuresis is a common pediatric problem for which, no unique therapy has been suggested. The conventional therapy is effective, but fails in some cases. So, many parents try complementary medicine. Therefore, this study attempted to find if rubbing coconut oil is effective on improving enuresis.

**Materials and Methods:**

This double-blinded randomized clinical was conducted on 120 children aged 6 to 14 years with mono symptomatic nocturnal enuresis, from 2018 to 2019 in Yazd, Iran. The drug and placebo groups applied 6 drops of the coconut and paraffin oil topically on the suprapubic, sacral and flanks areas one time per night, respectively. Urination pattern was daily recorded for a period of 8 weeks by parents, and after one year, they were asked for any improvement by phone call.

**Results:**

The mean frequency of enuresis at the first, second, fourth, and eighth week was lower in the intervention group (p<0.001); this difference between the groups remained after one year. Moreover, there was no side effect requiring any medical attention.

**Conclusion:**

Rubbing coconut oil is effective on improving symptom of primary mono symptomatic enuresis if applied every night for 4 weeks on suprapubic, sacral and flanks areas. This may be related to anticholinergic effect of the oil but its persistent effect for longer time after the end of application period, needs to be investigated in other studies.

## Introduction

Enuresis is considered a common pediatric problem, and more than 15% of children have enuresis during the first five years of age, but it decreases to 1% by fifteen years of age.

By definition, enuresis is the wetting of clothes or bedding with urine during the night for a period of at least 3 consecutive months in children over 5 years old.

Mono symptomatic nocturnal enuresis (MNE) can be defined as follows: enuresis in children with no other lower urinary tract symptoms, or a history of bladder dysfunction, comprising approximately 80% of primary enuresis (Nevéus et al., 2006 ▶). 

The exact etiology of MNE is not clear yet. It is thought that a single factor or combination of them may contribute to the condition. The factors listed for people with enuresis are as follows: male gender, history of enuresis in the family, some genes (12q, 13q, 8q), family’s education and economic status are considered.

 Diseases like constipation, UTI (urinary tract infection) and allergy have been found to be associated with nocturnal enuresis (Nørgaard et al., 1997 ▶). Normal bladder micturition and continence depend on a complex interaction among cholinergic, adrenergic, nitrergic, and peptidergic systems in the bladder wall. Cholinergic system acts on different Muscarinic receptors (M1-4) to modulate contraction in the bladder, and by definition, anticholinergic medication decreases its contractility (Sellers and Chess-Williams, 2012 ▶). 

Conventional treatments (alarm, vasopressin, and medication like anti-cholinergic agents) are based on these concepts that three factors play major roles in MNE pathology , these factors include higher urine production in the night, lower nocturnal bladder capacity with or without over activity of the bladder, and difficulty in arousal. Modalities of treatment are effective in the majority of cases; however, some cases are refractory to treatment and need a combination of the above-mentioned modalities, although relapse is a common problem with treatment caseation. Also, some parents are reluctant to long-term use of pharmacological drugs for their children or children cannot cope with therapy due to its side effects (Huang et al., 2011 ▶).

Above-mentioned facts make alternative therapy as a common practice in different regions of the world, due to their availability and affordability. Medicinal herbs are frequently used for MNE therapy (Huang et al., 2011 ▶; Motaharifard et al., 2020 ▶).

This study attempted to find if rubbing the bladder by coconut oil is effective on MNE. In this study, coconut was selected as a remedy, because in traditional Iranian medicine, coconut oil rubbing alleviates bladder problem by warming up the bladder (Nojavan et al., 2015 ▶; AghiliKhorasaniShirazi, 2014 ▶).

## Materials and Methods

The study was designed as a 2-arm, double blind, randomized, placebo-controlled clinical trial using a parallel design with a 1:1 allocation ratio. In this study, 120 children aged 6 to 14 years who referred to different Clinics of Shahid Sadoughi University of medical science between May 2018 and November 2019 with a clinical diagnosis of mono symptomatic nocturnal enuresis (MNE), were included, if their parents were willing to participate. Children with other lower urinary tract symptoms including increased (>8 times/day) or decreased (<3times/day) voiding frequency, urgency, hesitancy, straining, a weak stream, holding maneuvers, difficult emptying, post micturition dribble, and genital or lower urinary tract pain or constipation were excluded from the study. Any child who had developed UTI, trauma or urological problem during the course of treatment was also excluded from the study.

The sample size was calculated by a statistician considering a one-sided significance level of 0.05, a power of 0.80, and an alpha of 0.05 based on enuresis (p1 50% and p2 80%). Also, the minimum sample size required was calculated 52 participants per each group. Five percent was our estimation for the loss of follow up (Sharifi et al., 2017 ▶).

**Intervention**

Coconut oil extracted from fresh *Cocos nucifera* (Herbarium number: SSU0067) using cold press method was freely delivered for four weeks application. The parents were instructed to use 6 drops of the coconut oil topically on the suprapubic, sacral and flanks areas of their child once per night. Also, paraffin was selected as placebo for the control group participants who received 6 drops of oil one time per night in the mentioned areas similar to the intervention group. The participants of both groups fill out the form for daily urination pattern and enuresis every day.

**Randomization, blinding, and allocation concealment**

The physicians, researchers, and statisticians were blinded to the allocation of the children. The same shape and size of the drug and placebo containers were selected with a similarity in oils color, but the odor was different. The randomized list was generated using Microsoft Excel with a block randomization method, which divided these 120 children as 60 participants in each group.

**Outcomes**

Children were evaluated in terms of the frequency of enuresis prior to and following each week for 8 weeks of the intervention, and then, one year after the intervention. The number of the participants with any observed or reported adverse events was also registered for comparison.

**Statistical analysis**

Descriptive data are presented as means and standard error of means in quantitative data, and as percentage in case of qualitative data. Student t test, paired t test, and w2 test were used for statistical comparison.

**Ethical considerations**

The study protocol was in compliance with the Declaration of Helsinki (1989 revision), and it was approved by the Local Medical Ethics Committee of Shahid Sadoughi University of Medical Science, also, this study was provided with the following reference number: the trial IR.SSU.REC.1395.197 was registered in the Iranian Clinical Trials Registry (Registration ID: IRCT20170423033605N2).

## Results

**Figure 1:**
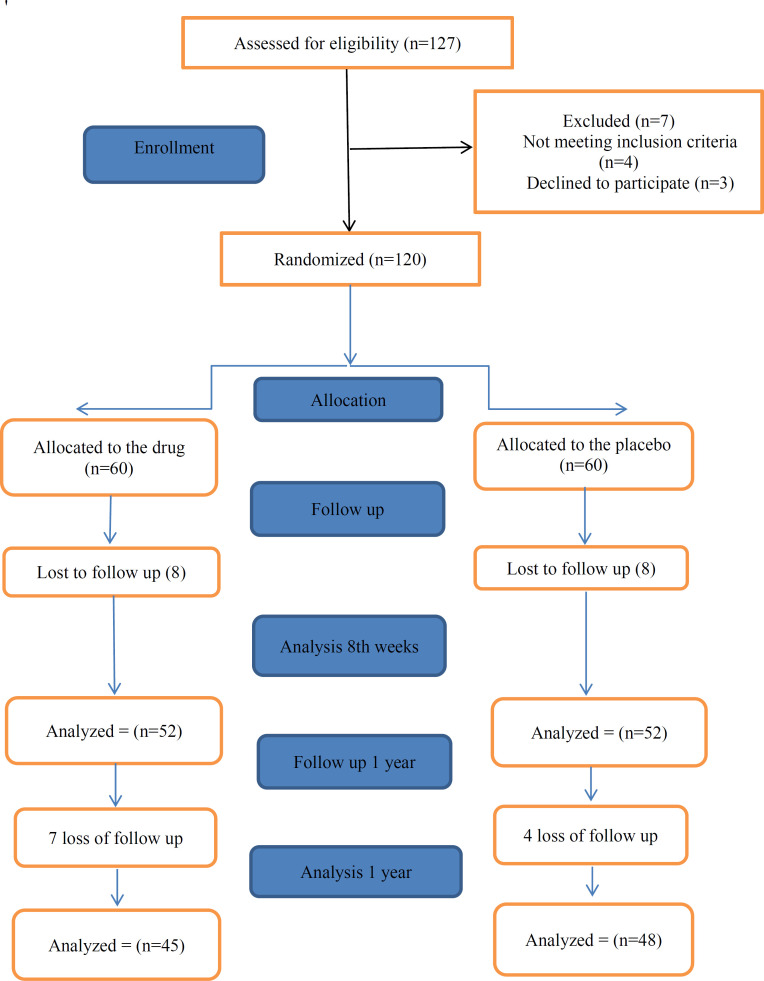
Flow chart of clinical trial of cocoanut oil massage for mono symptomatic nocturnal enuresis

From May 2018 to November 2019, a total of 127 children were known as eligible to receive the trial drug or placebo. Figure 1 shows a flow chart revealing the detailed descriptions of the patients’ enrolment, randomization, and follow up for one year.

Figures 1 demonstrate data from our cases in 8 week and one year follow up. 

In this study, 71 boys (68%) and 33 girls (32%) were included; of them, 49 (75%) boys and 14 (25%) girls were in the drug group and 32 (62%) boys and 20 (38%) girls were in the placebo group (p =0.2). The mean age of the drug group was 8.1±1.9 years old, while it was 8.0±2.1 years old in the placcebo group (p=0.63). The mean frequency of enuresis significantly decreased in the intervention group in the first, second, 4th and 8th weeks when compared with the base scores (Table 1). The mean frequency of enuresis in the first, second, 4th and 8th weeks was statistically significantly lower in the intervention group (p<.001) (Table 2).

**Figure 2:**
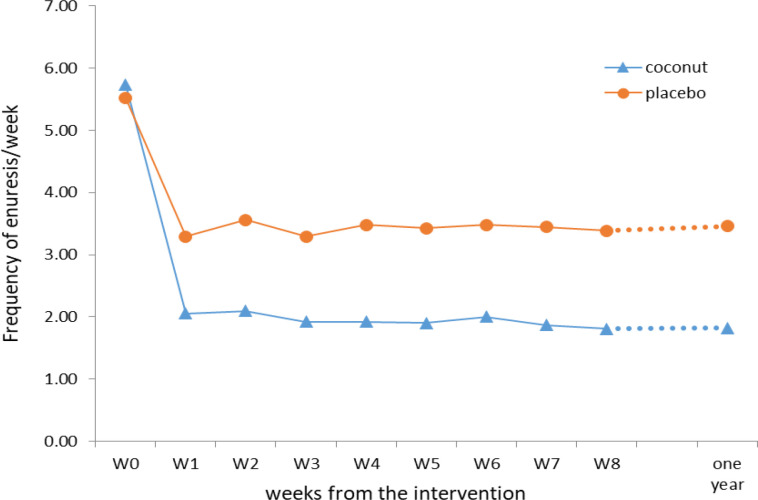
Pattern of improvement in the two groups by over time

There was no difference between the two genders in recovery (p=0.2). One year after the intervention, the majority of our drug group were dry, which was statically significant compared to the placebo (p<0.001 Figure 2). In terms of numbers, 24 drug group and 17 subjects in the placebo group experienced complete improvement, and 5 and 14 subjects had no improvement at all in the drug and placebo groups, and relapse was seen in 4 drug and 7 placebo respectively. 

There was no report of any adverse event in the study groups, but one had mild rash and itching. However, 7 parents complained of the greasy nature of the drug in the placebo group.

**Table 1:** The mean frequency of enuresis before the intervention compared with the first, second, third, fourth, eighth weeks and one year after the intervention

Paired differences						
				95% Confidence interval of the difference		
Groups		Mean differences	SEM	Lower	Upper	*P* Value^a^
Coconut	Base-1st week	3.673	0.304	3.062	4.284	<.001
	Base-2nd week	3.635	0.314	3.004	4.266	<.001
	Base-4th week	3.808	0.314	3.177	4.439	<.001
	Base-8th week	3.923	0.337	3.246	4.600	<.001
	Base-First year	3.844	0.366	3.106	4.583	<.001
Placebo	Base-1st week	2.231	0.299	1.630	2.831	<.001
	Base-2nd week	1.962	0.292	1.376	2.547	<.001
	Base-4th week	2.038	0.306	1.425	2.652	<.001
	Base-8th week	2.135	0.319	1.494	2.775	<.001
	Base-First year	2.083	0.322	1.436	2.731	<.001

Abbreviation: SEM, standard error of mean^a^Paired *t* test

**Table 2:** Comparison of the mean frequency of enuresis in the coconut and placebo groups

	Groups (mean±SEM)				95% Confidence interval of the difference		
Frequency of enuresis	Coconut	Placebo		Difference	Lower	Upper	p Value^a^
Baseline	5.73±0.24	5.52±0.26		0.212	-0.485	0.908	0.548
After 1 week	2.06±0.31	3.29±0.35		-1.231	-2.164	-0.297	0.010
After 2 weeks	2.10±0.31	3.56±0.36		-1.462	-2.404	-0.519	0.003
After 3 weeks	1.92±0.32	3.29±0.37		-1.365	-2.332	-0.398	0.006
After 4 weeks	1.92±0.30	3.48±0.37		-1.558	-2.503	-0.612	0.001
After 5 weeks	1.90±0.32	3.42±0.37		-1.519	-2.491	-0.547	0.003
After 6 weeks	2.00±0.31	3.48±0.36		-1.481	-2.419	-0.543	0.002
After 7 weeks	1.87±0.32	3.44±0.36		-1.577	-2.540	-0.614	0.002
After 8 weeks	1.81±0.31	3.38±0.36		-1.577	-2.532	-0.622	0.001
After one year	1.82±0.34	3.46±0.37		-1.636	-2.650	-0.622	0.002

Abbreviation: SEM, standard error of mean^a^Student's* t *test

## Discussion

This study tried to evaluate the efficacy of topical use of coconut oil on the suprapubic, sacral and flanks areas one time per night for the treatment of MNE. The results showed higher efficacy of coconut oil compared with placebo as the mean frequency of nocturia was significantly lower at the first week of coconut oil application compared to the placebo group, and this effect lasted for one year.

No human study has investigated the effect of coconut oil on bladder contraction, but an animal* and in vitro* study showed this inhibitory effect, experienced on mice gut showed coconut extract inhibits smooth muscle contraction of intestinal rat against acetylcholine. (Igboabuchi, 2010 ▶). This observation may explain the pharmacological role of coconut oil in alleviation of enuresis in our drug group. Remaining the therapeutic effect after stopping oil massage for a year requires more studies to confirm our finding. 

Traditional Persian medicine believes that the cold and wet nature of bladder sphincter is the main reason of primary enuresis (Igboabuchi, 2010 ▶). However, in deep sleeper brain distemperment may also play a role, prescription of herbs with opposite effects (warm and dry temperament) is considered in the traditional medicine for alleviating MNE (Igboabuchi, 2010 ▶). Also, a similar concept exists in Korean traditional medicine (Lee et al., 2018 ▶). Sharifi et al. applied chamomile oil and indicated similar results during 6 weeks, but they did not follow the cases for longer time (Sharifi et al., 2017 ▶). Few studies investigated herbs and enuresis, and data in spite of wide spread use of herbs for the treatment of enuresis, is scarce (Huang et al., 2011 ▶; Jaradat et al., 2017 ▶; Ma et al., 2017 ▶)

Coconut oil has been recommended for bladder problem in Persian medicine, and it is also prescribed in Turkish traditional medicine (Yıldırım et al., 2016 ▶). In addition, it is recommended by some commercial practitioner alone or in combination with other essential oils to treat enuresis (Pinterest, 2020 ▶).

We reported some findings in this study that were unusual and need to be mentioned. Accordingly, firstly, both drug and placebo groups showed improvement during the first two weeks, and then had a plateau state that is contrary to other studies, which reported that improvement rates gradually increased in the intervention group over time. Secondly, it is often observed in any modality of treatment for enuresis that relapse takes place after stopping any intervention, in the study 4 weeks after stopping of oil rubbing, our response rate did not change significantly (except in 4 cases). We lost 11 cases in one year of follow up, so interpretation would be difficult in a worst-case scenario. We expected 10% annual improvement with no intervention in MNE, and we did not observe such improvement in placebo group. The reasons may be due to the numbers that we lost in the follow up. The first observation may be explained by other innervation conducted on both groups like to chart urination, more parental attention, and oil massage. Massage and chiropractic care have some effects that are not considered certain in reviews yet (Clar et al., 2014 ▶; Bronfort et al., 2010 ▶; Instebø and Lystad, 2016 ▶).

Our limitation in this study was the absence of a standard treatment arm in the study. So, future research with a higher methodological quality is suggested, and the use of systemic formulation of coconut oil in comparison with the conventionally used intervention would be helpful

The findings of this study show that the topical use of coconut oil for 4 weeks can decrease enuresis at the first 8 weeks and longer follow up for one year showed its effect persisted in majority of drug group. Response took place at the first weeks, and its effect lasted for a year that needs to be confirmed in further studies. Our findings show that rubbing coconut oil may be considered a complementary method for MNE therapy with no side effect.

## Conflicts of interest

The authors have declared that there is no conflict of interest.
